# Emdogain-Regulated Gene Expression in Palatal Fibroblasts Requires TGF-βRI Kinase Signaling

**DOI:** 10.1371/journal.pone.0105672

**Published:** 2014-09-08

**Authors:** Alexandra Stähli, Dieter Bosshardt, Anton Sculean, Reinhard Gruber

**Affiliations:** 1 Department of Periodontology, School of Dental Medicine, University of Bern, Bern, Switzerland; 2 Laboratory of Oral Cell Biology, School of Dental Medicine, University of Bern, Bern, Switzerland; 3 Robert K. Schenk Laboratory of Oral Histology, School of Dental Medicine, University of Bern, Bern, Switzerland; Ohio State University, United States of America

## Abstract

Genome-wide microarrays have suggested that Emdogain regulates TGF-β target genes in gingival and palatal fibroblasts. However, definitive support for this contention and the extent to which TGF-β signaling contributes to the effects of Emdogain has remained elusive. We therefore studied the role of the TGF-β receptor I (TGF-βRI) kinase to mediate the effect of Emdogain on palatal fibroblasts. Palatal fibroblasts were exposed to Emdogain with and without the inhibitor for TGF-βRI kinase, SB431542. Emdogain caused 39 coding genes to be differentially expressed in palatal fibroblasts by microarray analysis (p<0.05; >10-fold). Importantly, in the presence of the TGF-βRI kinase inhibitor SB431542, Emdogain failed to cause any significant changes in gene expression. Consistent with this mechanism, three independent TGF-βRI kinase inhibitors and a TGF-β neutralizing antibody abrogated the increased expression of IL-11, a selected Emdogain target gene. The MAPK inhibitors SB203580 and U0126 lowered the impact of Emdogain on IL-11 expression. The data support that TGF-βRI kinase activity is necessary to mediate the effects of Emdogain on gene expression in vitro.

## Introduction

Emdogain consists of enamel matrix derivatives and the vehicle propylene glycol alginate (Institut Straumann, Basel, Switzerland) [1]. The local application of Emdogain has been shown to support skin wound healing [2], [3]. The ability of Emdogain to stimulate soft tissue regeneration has prompted the combination of Emdogain with palatal subepithelial connective tissue to enhance the healing process [4]–[6]. Emdogain has also been successfully used for regenerative treatment of various periodontal lesions such as intrabony defects, class II furcations, and recessions [7]–[9]. However, the cellular and molecular mechanisms allowing Emdogain to support tissue regeneration have not been clarified so far.

In vitro studies support the assumption that Emdogain directly targets cells that are involved in wound healing. For example, Emdogain modulates the formation of extracellular matrix and modulates the differentiation of mesenchymal cells [10], [11]. Emdogain can be taken up by periodontal ligament fibroblasts [12] and can change the mitogenic activity of cells [13]. Among the genes that are expressed in response to Emdogain are cytokines [14]. The in vitro cellular responses to Emdogain have been summarized recently [1], [15].

Microarray analyses have provided further insight into the complex cellular response to Emdogain, as was reported for periodontal ligament fibroblasts [16], [17], osteoblast-like cell lines (MG-63) [18], marrow stromal cells [19], and epithelial cell lines [20]. Recently, whole genome gene expression profiling with gingival and palatal fibroblasts has been performed, revealing numerous genes such as IL-11 that are typically regulated by TGF-β [21]. It is thus possible that at least some of the cellular responses to Emdogain involve TGF-β activity.

This assumption is supported by observations of neutralizing antibodies against TGF-β substantially reducing the impact of Emdogain on cell signaling [22], connective tissue growth factor expression [23] or proliferation [24]. Also, SB431542, a pharmacologic inhibitor of the TGF-β type I receptor (TGF-βRI) kinase, suppresses the in vitro effect of Emdogain on adipogenesis [11] and osteoclastogenesis [25], but also of calcium channel blockers [26]. It is thus supposed that TGF-β type I receptor is required to mediate Emdogain-induced gene expression in palatal fibroblasts. The key question is, to what extent?

TGF-β signaling controls multiple cellular responses, including cell growth and differentiation [27]. Ligand binding causes the type I and type II receptors to form a complex that initiates activation of the cytoplasmic kinase, which in turn phosphorylates Smad2/3. A Smad (small mothers against decapentaplegic) complex is formed that controls the expression of target genes in the cell nucleus. Ligand binding can also activate a non-canonical pathway, for example, mitogen-activated protein kinase signaling including ERK, JNK, and p38 MAPK pathways. Emdogain was reported to activate Smad2/3 [11], [28] and MAPK pathways [28], further supporting the evidence that Emdogain stimulates TGF-β signaling.

Existing knowledge led us to ask to what extent Emdogain exerts its cellular responsiveness via TGF-β signaling. To answer the question, we determined the gene expression profile of palatal fibroblasts exposed to Emdogain with and without the presence of the TGF-βRI kinase inhibitor SB431542. We found that cells exposed to SB431542 were completely shielded from the Emdogain-induced gene expression.

## Materials and Methods

### Isolation of fibroblasts and exposure to Emdogain

Human palatal fibroblasts were prepared from tissue grafts removed during periodontal surgery. Palatal grafts were harvested from three individual patients each. Tissue explants were cultivated in Dulbecco’s Modified Eagle Medium supplemented with 10% fetal calf serum (PAA Laboratories, Linz, Austria) and antibiotics. Fibroblasts that grew out from the explants and had not undergone more than five passages were used for the experiments. Palatal fibroblasts were plated at 30,000 cells/cm^2^ and incubated for 24 hours with Emdogain (Institut Straumann AG, Basel, Switzerland) at 100 µg enamel matrix derivative per ml or serum-free medium alone.

### Ethics statement

Fibroblasts were retrieved from patients undergoing periodontal surgery after signed informed consent and approval by the Ethics Committee of the University of Bern.

### Modulation by pharmacologic inhibitors and antibodies

Pharmacologic inhibitors for the TGF-βRI kinase were SB431542 (Calbiochem, Merck, Billerica, MA), activin receptor-like kinase-5 ALK5 Inhibitor I (LY-364947; Enzo Life Sciences AG, Lausen, Switzerland), and ALK5 Inhibitor II (2-(3-(6-Methylpyridin-2-yl)-1H-pyrazol-4-yl)-1,5-naphthyridine; Enzo), all at 10 µM. Pharmacologic inhibitors for the three main MAPK signaling pathways were U0126, SB203580, and SP600125, all at 10 µM (Santa Cruz Biotechnology, SCBT; Santa Cruz, CA). The smad3 inhibitor SIS3 was obtained from Calbiochem. The TGF-β pan specific polyclonal Ab, AB-100-NA was obtained from R&D Systems Inc. (Minneapolis, MN). The bone morphogenetic protein (BMP) type I receptor kinase inhibitors, dorsomorphin (Sigma, St. Louis, MO) and LDN193189 (Cayman, Ann Arbor, MI) were used at 10 µM and 1 µM, respectively. The impact of the TGF-βRI kinase inhibitors on cell viability was determined by the conversion of MTT into formazan crystals [29] and Nuclear-ID Red/Green cell viability reagent (Enzo Life Sciences, Inc., Farmingdale, NY).

### Microarray analysis

Total RNA was isolated using the High Pure RNA Isolation Kit (Roche Applied Science, Rotkreuz, Switzerland). RNA quality was determined using the Agilent 2100 Bioanalyzer (Agilent Technologies, Santa Clara, CA, USA). Microarray analysis was performed using the Human GE 4×44K V2 Microarray Kit with SurePrint Technology (Illumina Inc., San Diego, CA, USA) recognizing mRNA and long intergenic non-coding RNA (lincRNA). Array image acquisition was performed with the Agilent G2505B Microarray Scanner and Feature Extraction software version 9.5 (Agilent). Data files were analyzed by GeneSpring GX 7.3.1. Microarray analysis was performed at Arrows Biomedical Deutschland GmbH (Münster, Germany).

### qRT-PCR analysis and immunoassay

For validation of the microarray results, one gene from the gene list was selected for qRT-PCR analysis. Reverse transcription (RT) was performed with Transcriptor Universal cDNA Master (Roche) and PCR was done with the FastStart Universal Probe Master Rox (Roche) on a 7500 Real-Time PCR System (Applied Biosystems, Carlsbad, CA, USA). Probes were designed with the online Universal ProbeLibrary System (Roche): IL11 Forw: GGA CAG GGA AGG GTT AAA GG, Rev: GCT CAG CAC GAC CAG GAC; SNAI1 Forw: GCT GCA GGA CTC TAA TCC AGA, Rev: ATC TCC GGA GGT GGG ATG; SNAI2 Forw: TGG TTG CTT CAA GGA CAC AT, Rev: GCA AAT GCT CTG TTG CAG TG; CTGF Forw: CCT GCA GGC TAG AGA AGC AG, Rev: TGG AGA TTT TGG GAG TAC GG. TGF-β Forw: ACT ACT ACG CCA AGG AGG TCA C, Rev: TGC TTG AAC TTG TCA TAG ATT TCG. The mRNA levels were calculated by normalizing to the housekeeping gene beta actin using the ΔΔCt method. The immunoassay for human IL-11 was obtained from Enzo Life Sciences.

### Transfection with siRNA

TGF-β1 siRNA, mock siRNA and the transfection agent were purchased from SCBT (Santa Cruz, CA). The transfection protocol was followed according to the instructions of the manufacturer. Inhibition efficacy was determined by basal expression control of the TGF-β1-regulated genes SNAI1, SNAI2, and CTGF. Transfected cells were exposed to Emdogain at 100 µg/ml in serum-free medium for 24 hours. Gene expression analysis was performed targeting IL-11.

### Western blot analysis

Palatal fibroblasts were serum-starved and then treated with Emdogain for 30 minutes. Cell extracts were separated by SDS-PAGE and transferred onto nitrocellulose membranes. Binding of the antibody raised against phospho-smad3 (both Cell Signaling Technology, Danvers, MA, USA) and β-actin (SCBT) were detected with the appropriate secondary antibody directly labeled with near-infrared dyes (Invitrogen) and detected with the appropriate imaging system (LI-COR Biosciences).

### Functional annotation and molecular network analysis

To assign biological meaning of the subset of genes, Gene Ontology screening was performed (GO; DAVID. (david.abcc.ncifcrf.gov/home.jsp): GOTERM_BP_FAT (biological process), GOTERM_MF_FAT (molecular function), GOTERM_CC_FAT (cellular component), and KEGG Pathway (www.genome.jp/kegg/pathway.html). DAVID calculates a modified Fisher’s Exact p value to demonstrate GO or molecular pathway enrichment. P values less than 0.05 after Benjamini multiple test correction were considered strongly enriched in the annotation category.

### Statistical analysis

The numbers of differentially expressed transcripts in the microarray data were identified (≥10-fold change; p<0.05) under the analysis of variance and post hoc Benjamini–Hochberg false discovery rate correction for multiple tests. RT-PCR data were calculated with the paired T-test.

## Results

### Dose- and time-dependent responses of Emdogain on IL-11 expression

**Figure 1:**
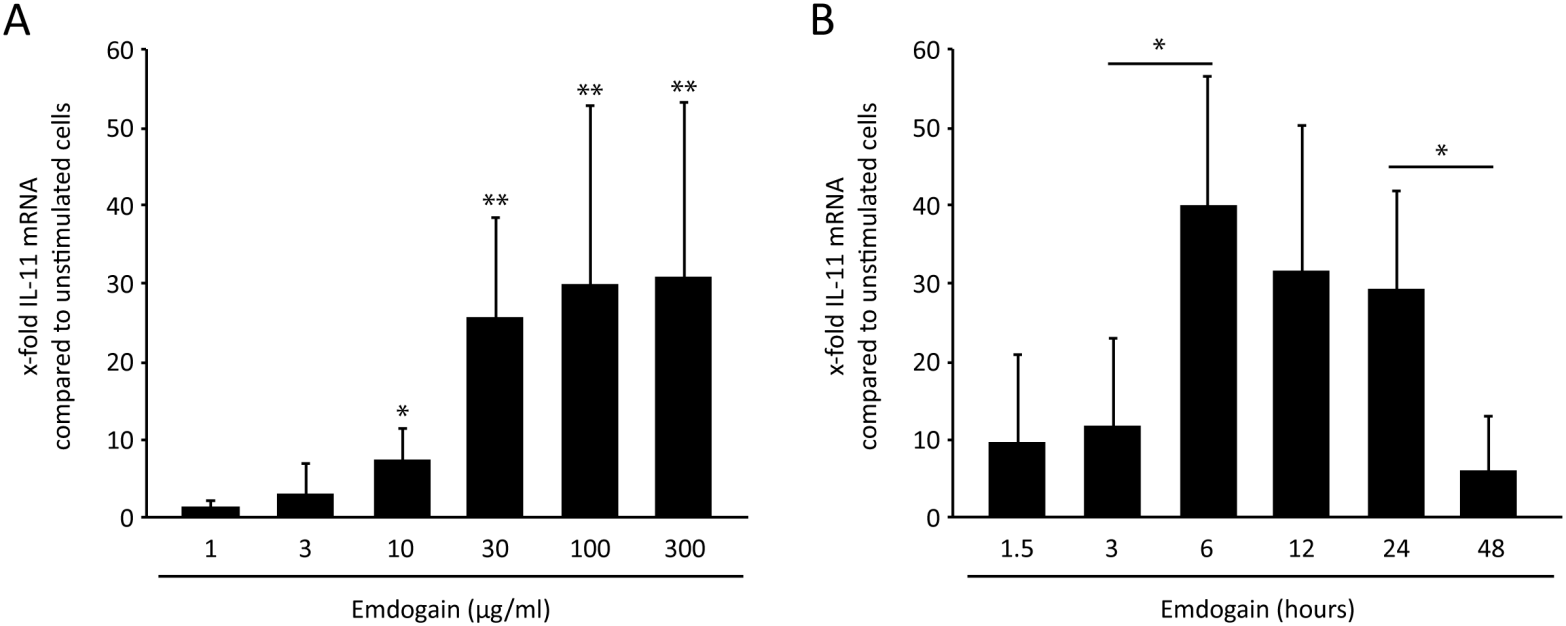
Dose- and time-response of Emdogain on IL-11 expression. Palatal fibroblasts were incubated with (A) various concentrations of Emdogain for 24 hours and for (B) various time-points with Emdogain at 100 µg/ml. RT-PCR was performed for IL-11 **P<0.01 compared to Emdogain control. The data represent two experiments with a total of three donors (n = 6).

**Figure 2:**
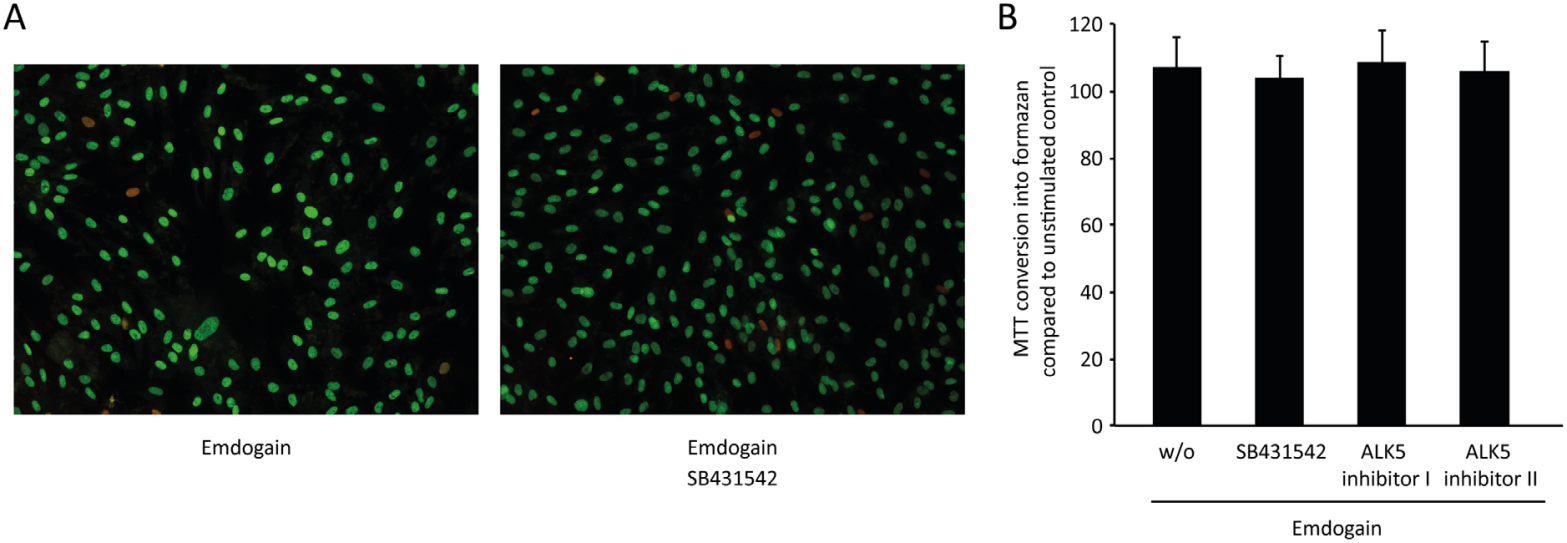
Viability is maintained when cells are exposed to TGF-β receptor I kinase inhibitors. Palatal fibroblasts were incubated with 100 µg/ml Emdogain or serum-free medium alone and the TGF-βRI kinase inhibitors SB431542, ALK5 Inhibitor I or ALK5 Inhibitor II. (A) MTT conversion assay shows that the presence of the three TGF-βRI kinase inhibitors had no significant impact on cell viability. (B) The nuclear-ID Red/Green cell viability assay confirms this finding as the distribution of viable green and dead red cells is not affected by SB431542. Experiments were performed three times.

To reveal the most suitable experimental conditions, palatal fibroblasts were incubated with various concentrations of Emdogain for 24 hours (Figure 1A), and with a single concentration of 100 µg/ml for various time-points (Figure 1B). Based on the previous findings [21], IL-11 was selected as an indicator gene for the effects of Emdogain in vitro. The dose-response curve confirmed the commonly used concentration of 100 µg/ml Emdogain to provoke a maximal increase of IL-11 expression and also the time-response curve supports the 24-hour incubation period as ideal for this experimental set-up, prior to the genome-wide microarray. To rule out any toxic effect of the TGF-βRI kinase inhibitor, palatal fibroblasts were incubated with Emdogain with and without SB431542 before life-dead staining (Figure 2A) and MTT assay (Figure 2B) were performed.

### TGF-β receptor I kinase inhibitor suppressed the expression of all Emdogain-regulated genes

To study the role of the TGF-βRI kinase to mediate the effect of Emdogain in vitro, isolated palatal fibroblasts from three donors were exposed to Emdogain with and without the inhibitor SB431542 and a genome-wide microarray was performed. The gene expression profiles of palatal fibroblasts revealed that Emdogain greatly changed the expression of 39 coding genes (>10-fold, p<0.05, Table 1), similar to what we have recently reported [21]. Strikingly, SB431542 completely abolished the expression of all Emdogain-regulated genes (Table 1). Also five non-coding Agilent Gene IDs that were regulated by Emdogain failed to do so in the presence of SB431542 (Table 2). Together these data suggest that the respective fibroblastic response to Emdogain essentially requires the TGF-βRI kinase activity.

**Table 1:** SB431542 suppressed the expression of coding genes regulated by Emdogain.

	Gene ID	Change		Gene Name
		wo/E	wo/ESB43	
1.	A_23_P39955	17.0	−1.1	actin, gamma 2, smooth muscle, enteric
2.	A_33_P3310929	11.5	−1.2	ADAM metallopeptidase domain 12
3.	A_23_P14083	13.1	−1.3	adhesion molecule with Ig-like domain 2
4.	A_32_P105549	10.9	−1.2	annexin A8 (and like 1 & 2)
5.	A_33_P3385266	−10.1	1.2	ATP-binding cassette, sub-family C, member 6 pseudogene
6.	A_23_P125233	10.7	−1.1	calponin 1, basic, smooth muscle
7.	A_23_P151895	41.3	−1.3	cartilage intermediate layer protein, nucl. pyrophosphohydrolase
8.	A_23_P121695	10.0	1.0	chemokine (C-X-C motif) ligand 13
9.	A_23_P362191	−18.5	−2.1	chromosome 17 open reading frame 44 & 68
10.	A_23_P251043	12.1	−1.6	chromosome 20 open reading frame 39
11.	A_33_P3423854	10.0	−1.3	complement component 8, beta polypeptide
12.	A_23_P65518	10.2	−1.8	dapper, antagonist of beta-catenin, homolog 1 (Xenopus laevis)
13.	A_23_P46936	18.4	−2.5	early growth response 2
14.	A_32_P51237	197.3	−1.1	KN motif and ankyrin repeat domains 4
15.	A_33_P3283833	13.1	−1.7	forkhead box S1
16.	A_23_P396858	17.1	1.3	frizzled homolog 8 (Drosophila)
17.	A_23_P105251	18.9	−2.1	GLI family zinc finger 1
18.	A_32_P140489	12.8	1.0	growth differentiation factor 6
19.	A_24_P140608	26.3	−1.1	heparin-binding EGF-like growth factor
20.	A_33_P3243887	15.7	−1.6	interleukin 11
21.	A_33_P3260530	32.3	−1.2	KN motif and ankyrin repeat domains 4
22.	A_24_P827037	10.4	−1.4	leucine rich repeat containing 15
23.	A_23_P6771	26.8	1.2	LIM and cysteine-rich domains 1
24.	A_33_P3214334	12.4	1.9	lymphocyte antigen 6 complex, locus G6F
25.	A_33_P3312676	14.0	−1.3	myelin transcription factor 1
26.	A_33_P3246418	28.9	1.1	MyoD family inhibitor
27.	A_33_P3224324	15.7	1.6	NADPH oxidase 4
28.	A_23_P138194	26.2	−1.3	neutrophil cytosolic factor 2
29.	A_23_P151506	22.2	2.7	pleckstrin 2
30.	A_23_P210581	12.6	−1.2	potassium voltage-gated channel, subfamily G, member 1
31.	A_24_P413126	14.7	−2.2	prostate transmembrane protein, androgen induced 1
32.	A_33_P3369178	27.4	−2.1	proteoglycan 4
33.	A_24_P13041	12.9	2.5	rhotekin 2
34.	A_33_P3299510	10.2	−1.5	scleraxis homolog A (mouse); scleraxis homolog B (mouse)
35.	A_24_P48204	−18.2	1.1	secreted and transmembrane 1
36.	A_23_P106389	25.4	1.0	semaphorin 7A, GPI membrane anchor
37.	A_23_P434398	−10.8	1.2	taxilin beta
38.	A_32_P75264	−12.0	−1.6	transmembrane protein 26
39.	A_24_P226970	17.4	1.0	zinc finger protein 365

Palatal fibroblasts were exposed to Emdogain (E) with and without the inhibitor SB431542 (SB43) and a genome-wide microarray was performed. The table shows the genes with a coding sequence that are at least 10-fold changed by Emdogain. SB431542 completely abolished the expression of all Emdogain–regulated genes. The data represent the means of one experiment with cells from three individual donors.

**Table 2:** SB431542 suppressed the expression of non-coding sequence regulated by Emdogain.

	Agilent Gene ID	Change	
		wo/E	wo/ESB43
1.	A_23_P102681	14.7	−1.4
2.	A_23_P166779	20.6	−1.4
3.	A_24_P223018	−14.5	−2.0
4.	A_33_P3257518	15.0	−1.3
5.	A_33_P3402329	38.7	−1.6

Genome-wide microarray from palatal fibroblasts revealed genes with a non-coding sequence that are at least 10-fold changed by Emdogain (E). SB431542 (SB43) completely abolished the expression of all Emdogain–regulated genes.

### TGF-β receptor I kinase is crucial for IL-11 expression induced by Emdogain

**Figure 3:**
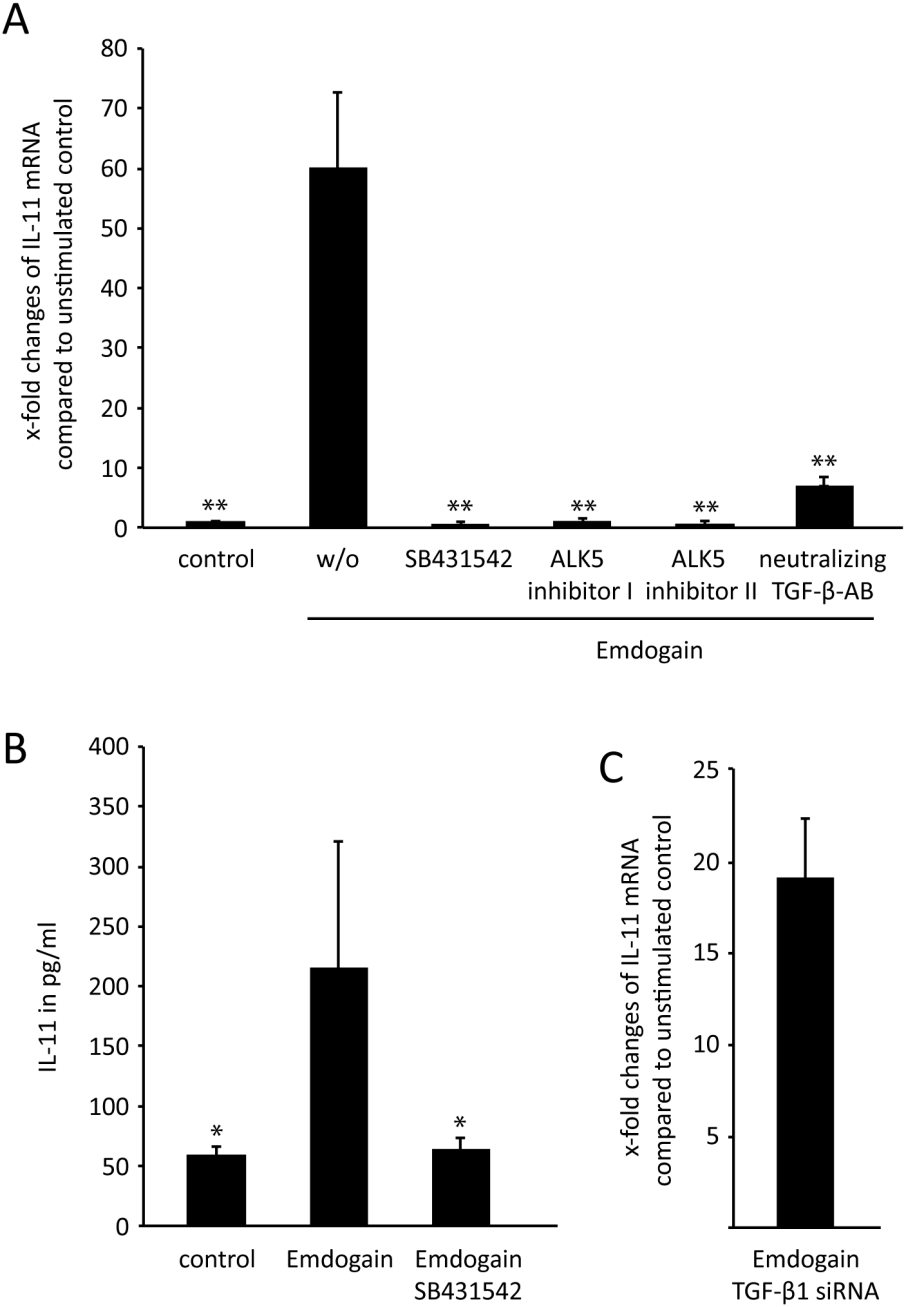
TGF-β receptor I kinase is crucial for IL-11 expression induced by Emdogain. Palatal fibroblasts were incubated with 100 µg/ml Emdogain or serum-free medium alone and the TGF-βRI kinase inhibitors SB431542, ALK5 Inhibitor I or ALK5 Inhibitor II and a neutralizing TGF-β pan-specific polyclonal antibody before RT-PCR was performed for IL-11 (A). The data represent 8 experiments including 4 donors. Immunoassay for IL-11 support the data at the protein level (2 experiments with 2 donors). **P<0.01 compared to Emdogain control (B). Palatal fibroblasts transfected with TGF-β1 siRNA and mock siRNA similarly respond to Emdogain by an increase IL-11 expression (C). The basal expression of TGF-β1-regulated genes SNAI1, SNAI2, and CTGF were around 50% decreased by TGF-β1 siRNA indicating that the transfection was effective (data not shown). The latter data represent one experiment with two donors (n = 2). Not shown is that Emdogain fails to change basal TGF-β1 expression.

In line with the microarray data, quantitative RT-PCR confirmed the strong increase of IL-11 when palatal fibroblasts were exposed to Emdogain (Figure 3A). Also in this setting, the TGF-βRI kinase inhibitor SB431542 completely abolished the impact of Emdogain on expression of IL-11 by palatal fibroblasts. Furthermore, two other inhibitors for TGF-β receptor I kinase (ALK5 Inhibitor I or II) also obliterated the Emdogain-stimulated increase of IL-11. Also the presence of a neutralizing TGF-β pan-specific polyclonal antibody reduced the Emdogain-induced IL-11 expression. The immunoassay further supports this observation by showing the decrease of IL-11 at the protein level (Figure 3B).

To understand a possible autocrine function of Emdogain, TGF-β expression was determined and siRNA-blocking experiments performed. Emdogain did not significantly change the basal TGF-β expression in the fibroblasts (data not shown). Moreover, blocking TGF-β1 translation by siRNA transfection did not alter the effect of Emdogain on IL-11 expression (Figure 3C). TGF-β1 siRNA decreased the basal expression of the respective target genes SNAI1, SNAI2, and CTGF (data not shown). Together, these findings support the role of TGF-βRI kinase in mediating effects of Emdogain on gene expression in vitro, independent of TGF-β1 produced by the palatal fibroblasts.

### Smad-3 and MAPK mediate the effect of Emdogain on IL-11 expression

**Figure 4:**
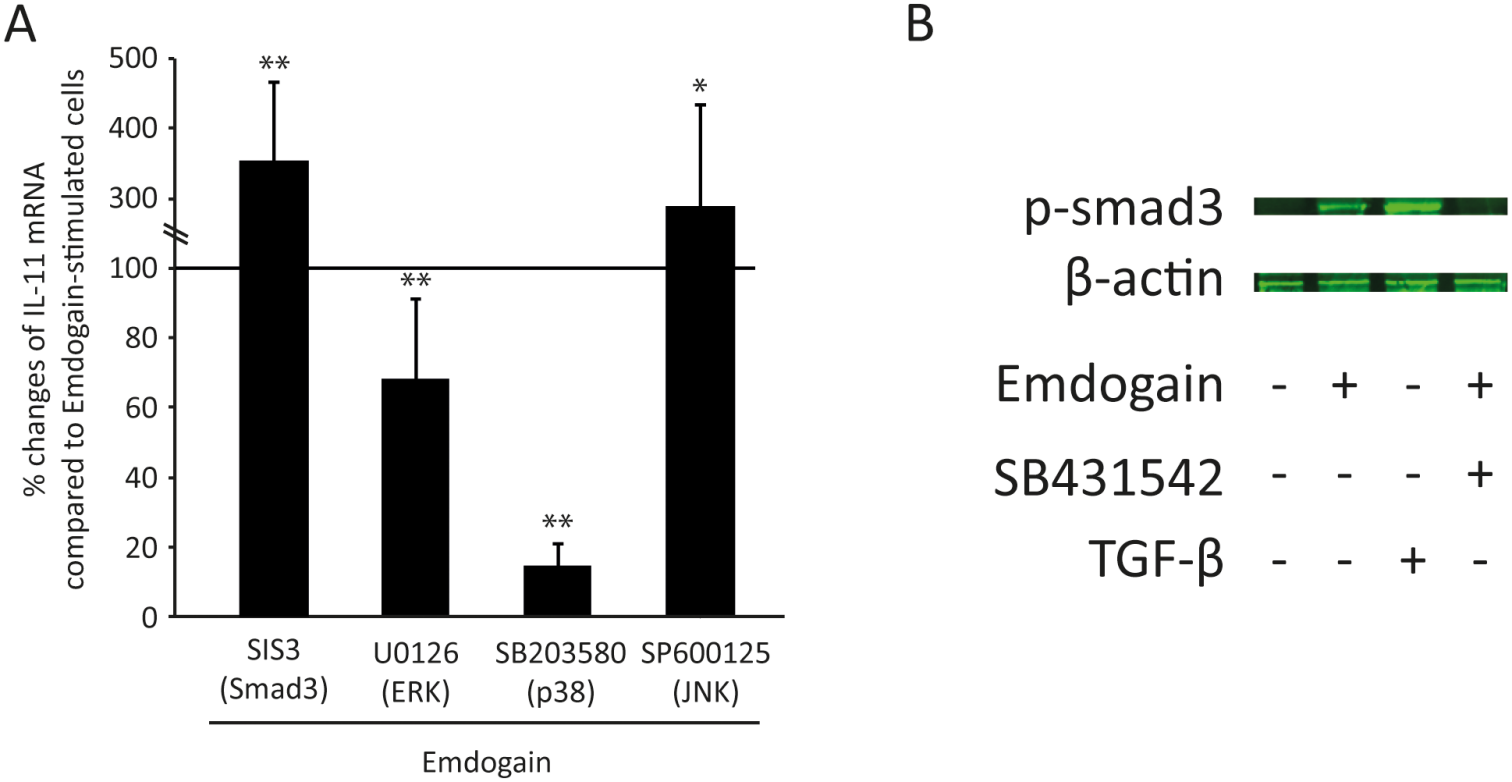
Smad-3 and MAPK mediate the effect of Emdogain on IL-11. Palatal fibroblasts were incubated with 100 µg/ml Emdogain or serum-free medium. The impact of the signaling inhibitors on IL-11 expression was determined by RT-PCR. Data represent 5 experiments with 3 donors.

We next sought to determine whether TGF-βRI kinase downstream signaling pathways involve canonical signaling by adding the smad-3 inhibitor SIS3. Surprisingly, SIS3 increased Emdogain-induced IL-11 expression (Figure 4A) even though Emdogain increased smad-3 phosphorylation in Western blot analysis (Figure 4B). These results suggest that also the non-canonical signaling pathway controls at least IL-11 expression. In support of this suggestion, blocking ERK and p38 with U0126 and SB203580, respectively, significantly reduced the effect of Emdogain on IL-11 expression (Figure 4A). Taken together, these results suggest that ERK and in particular p38 attenuate the expression of Emdogain-regulated IL-11 expression in palatal fibroblasts.

### BMP receptors do not mediate the effect of Emdogain on IL-11 expression

**Figure 5:**
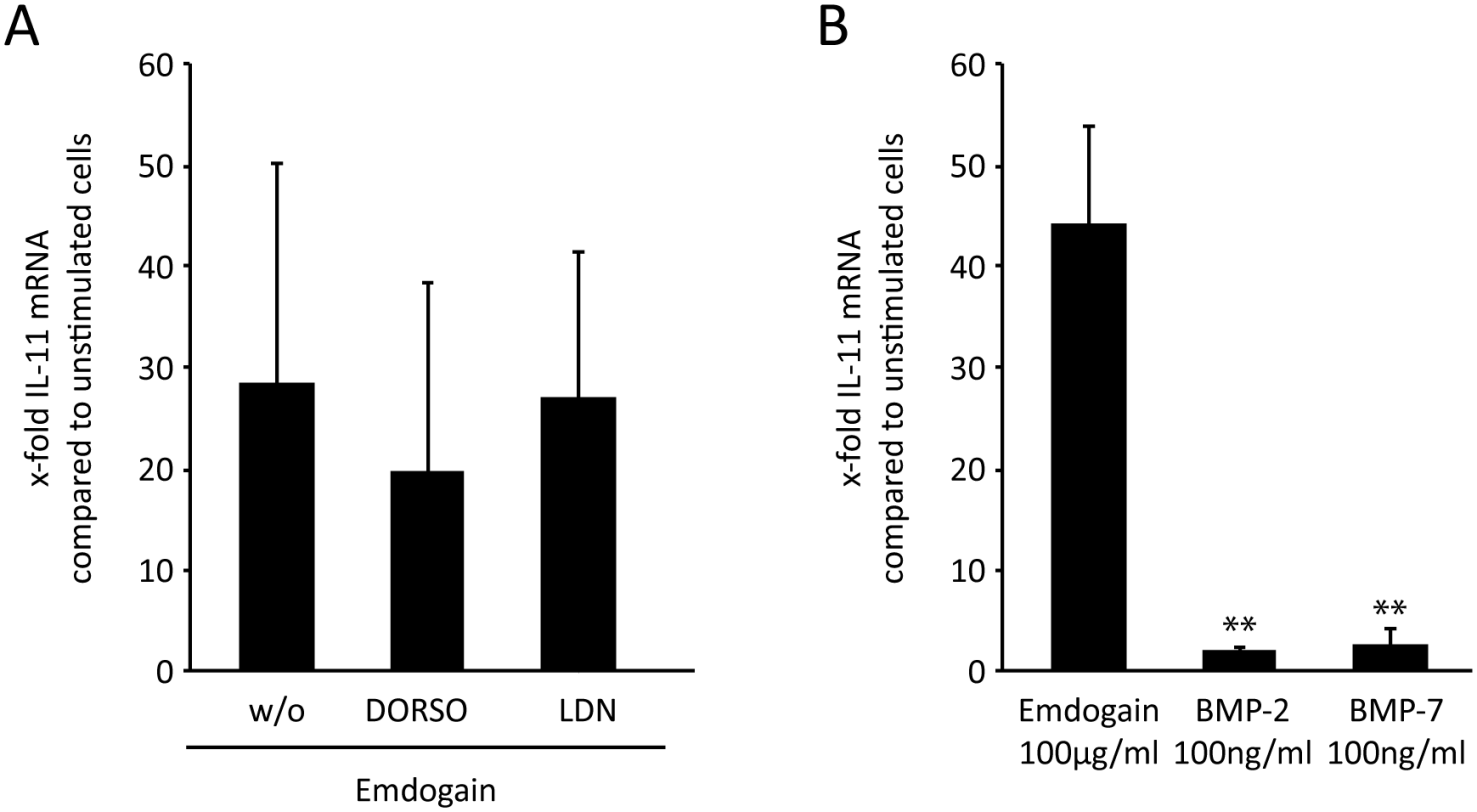
BMP receptors ALK2, ALK3, and ALK6 do not mediate the effect of Emdogain on IL-11 expression. Palatal fibroblasts were incubated with (A) 100 µg/ml Emdogain or serum-free medium alone and BMP type I receptor inhibitors dorsomorphin (DORSO; 10 µM) and LDN193189 (LDN; 10 µM). Palatal fibroblasts were also incubated with (B) recombinant human BMP-2 and BMP-7 (both 100 ng/ml). RT-PCR was performed for IL-11 **P<0.01 compared to Emdogain control. The data represent at least two experiments with cells from three donors (n = 6).

To further rule out that the effects of Emdogain are mediated via BMP receptors, palatal fibroblasts were incubated with the BMP type I receptor inhibitors dorsomorphin and LDN193189. Neither of the two inhibitors considerably reduced the Emdogain-induced IL-11 expression (Figure 5A). In line with this observation, recombinant BMP-2 and BMP-7 only had negligible effects on IL-11 expression (Figure 5B). Taken together, Emdogain effects on IL-11 expression occur independently of BMP type I receptors.

### Three domains of gene ontology: biological process, cellular component, and molecular function

As indicated in Tables 3–5, the 39 Emdogain-regulated coding genes were associated with 14 biological processes, six cellular components, and five molecular functions. The most relevant biological process was “response to wounding”, with 6 genes involved (p = 0.011), whereas the most relevant cellular component was “intrinsic to membrane”, with 17 genes involved (p = 0.27). The highest association for a molecular function was “cytokine activity” (p = 0.007), with chemokine ligand 13, growth differentiation factor 6, and Interleukin-11 being involved in this cluster. Gene ontology analysis underlined the complex cellular response of gingival and palatal fibroblasts to Emdogain in vitro.

**Table 3:** GO Analyze Functional Annotation Clustering.

GOTERM_BP_FAT			
Term	Count	%	Benjamini
Response to wounding	7	17.1	3.9E-1
Wound healing	4	9.8	8,7E-1
Immune response	6	14.6	9,4E-1
Coagulation	3	7.3	9,2E-1
Blood coagulation	3	7.3	9,2E-1
Hemostasis	3	7.3	8,9E-1
Superoxide anion generation	2	4.9	8,9E-1
Regulation of body fluid levels	3	7.3	9,3E-1
Defense response	5	12.2	9,4E-1
Superoxide metabolic process	2	4.9	9,4E-1
Regulation of protein amino acid phosphorylation	3	7.3	9,3E-1
Positive regulation of cell proliferation	4	9.8	9,4E-1
Regionalization	3	7.3	9,4E-1
Regulation of transcription from RNA polymerase II promoter	5	12.2	9,5E-1

**Table 4:** GO Analyze Functional Annotation Clustering.

GOTERM_CC_FAT			
Term	Count	%	Benjamini
Extracellular space	7	17.1	9.9E-2
Extracellular region part	7	17.1	2,4E-1
Plasma membrane	14	34.1	2,6E-1
Intrinsic to membrane	17	41.5	3,4E-1
Extracellular region	9	22.0	3,3E-1
Integral to membrane	16	39.0	3,8E-1

**Table 5:** GO Analyze Functional Annotation Clustering.

GOTERM_MF_FAT			
Term	Count	%	Benjamini
Calcium-dependent phospholipid binding	3	7.3	1.3E-1
Cytokine activity	4	9.8	3.2E-1
Oxidoreductase activity, acting on NADH or NADPH	2	4.9	5.4E-1
Growth factor activity	3	7.3	6.8E-1
Phospholipid binding	3	7.3	6.7E-1

## Discussion

Based on studies using antibodies [22]–[24] or pharmacologic inhibitors for the TGF-βRI kinase [11], [25], it has been suggested that Emdogain exerts, at least in part, its in vitro effects via a TGF-β-like activity. These data appear to indicate that TGF-β signaling may play an important role in the cellular responses caused by Emdogain in vitro. In support of this assumption, the genetic evidence presented in this study demonstrates that TGF-βRI kinase is fundamental to mediate Emdogain effects on gene expression in palatal fibroblasts. Our finding that in the presence of SB431542, none of the 39 Emdogain-regulated genes reaches the level of significance, greatly supports the concept that TGF-βRI kinase is critically involved in mediating palatal fibroblast responses to Emdogain.

Microarray analyses, consistent with our approach, have shown the complex genetic response of mesenchymal [16]–[19], [21] and epithelial cells [20] to Emdogain in vitro. Taken together, these studies revealed the spectrum of genes regulated by Emdogain – some of which are typical TGF-β-regulated genes; including the cytokine IL-11 [30]. Other examples are transcription factors such as NOX4 [31] and PPARG [32]. These reports, along with the finding that Emdogain demonstrates TGF-β-like activity [11], [22]–[25], support the hypothesis that Emdogain requires TGF-β receptor signaling to mediate the changes in gene expression. Our data showing that blocking TGF-βRI kinase in fibroblasts completely suppressed the response of cells to Emdogain corroborates this concept.

IL-11, a pleiotropic cytokine of the interleukin-6 type family [33], has been chosen as the “indicator” gene to investigate down-stream TGF-β signaling pathways, as reported for lung fibroblasts [30], periodontal ligament and gingival fibroblasts [34], and bone metastatic breast cancer cells [35]. A similar TGF-βRI-dependent increase in IL-11 expression was observed with another crude preparation of growth factors, bone conditioned medium [36]. Also other microarray studies demonstrate that Emdogain supports IL-11 expression in oral fibroblasts [16], [21]. In patients with aggressive periodontitis, IL-11 was decreased in periodontal pockets, pointing at a shift of the inflammatory equilibrium towards a more pro-inflammatory state [37]–[39]. Thus, it can be speculated that increasing IL-11 levels induced via TGF-βRI kinase might exert beneficial effects on tissue regeneration.

TGF-βRI, when activated, forms a complex with the type II receptor and phosphorylates Smad2/3 [27]. Consistent with previous evidence that Emdogain activates Smad2/3 signaling [11], [28], we confirmed here that Emdogain increased Smad3 phosphorylation and that SIS3, the Smad3-inhibitor, lowered Emdogain-induced Sema7a expression (data not shown). Surprisingly, SIS3 increased the impact of Emdogain on IL-11 expression. IL-11 expression depends on the canonical pathway in tumor cells [40], but also involves MAPK pathways [27], [41]. Also TGF-β1-stimulated LOX and VEGF expression involved Smad3 but also MAPK signaling [42] [43]. In line with this concept, we show that blocking of ERK and p38 lowered the impact of Emdogain on IL-11 expression, and Emdogain was already reported to cause activation of the respective kinases [22], [28]. It remains uncertain why SIS3 increased the impact of Emdogain on IL-11 expression.

BMP receptors might exert some activities of Emdogain [44], [45] via SMAD 1/5/8 [46]. Emdogain can increase the expression of BMP-2 [21], [47], but decrease the expression of BMP-4 in mesenchymal cells [48]. EMD can further induce the expression of all BMP receptors, particularly BMPR-2 [47]. Here, BMP inhibitors were used to delineate BMP effects from TGF-β and activin signaling [46], [49]. In the present study, dorsomorphin and LDN-193189 only marginally changed Emdogain-induced IL-11 expression. These findings support the assumption that BMP receptor signaling is not a central pathway in this in vitro setting. Further support for this assumption derives from our data showing that recombinant BMP-2 and BMP-7 do not change IL-11 expression in palatal fibroblasts. However, dorsomorphin can also inhibit TGF-β1 signaling through the ALK1 pathway [50] and prevent TGF-ß-induced CTGF and COL1A1 expression in synovial fibroblasts [51]. Taken together, the effects of Emdogain on IL-11 expression require ALK5 but not ACTR-I (ALK2), BMPR-IA (ALK3), or BMPR-IB (ALK 6). Besides IL-11, other Emdogain-regulated genes might depend on different downstream effects than ALK5 signaling, but this requires further studies.

The next question addressed was whether Emdogain-induced changes in gene expression are mediated via TGF-β1 produced by the palatal fibroblasts and thus by an autocrine mechanism. This question is based on the observation that Emdogain can stimulate TGF-β1 expression in oral fibroblasts [24], [52]. We have reason to suggest that this is not the case. First, Emdogain failed to significantly increase TGF-β1 expression in palatal fibroblasts. Second, Emdogain increased IL-11 expression within only 1.5 hours. Finally, transfection with siRNA against TGF-β did not alter the strong increase of IL-11 induced by Emdogain. Moreover, an immunoassay raised against TGF-β1 showed positive signals in Emdogain, which favors the hypothesis that at least the major impact on the regulation of IL-11 directly derives from Emdogain.

The Emdogain-regulated genes can be clustered by Gene Ontology into functional groups related to biological processes, cellular components, and molecular functions. For example, in line with previous studies [21], Emdogain affects “response to wounding”, “intrinsic to membrane”, and “cytokine activity”. However, in the present study the threshold with 10-fold changes in gene expression was higher than the previous 5-fold changes [21], thus only 39 compared to 106 genes went into Gene Ontology analysis. The overall interpretation of the Gene Ontology data, however, is that all the observed changes in biological processes, cellular components, and molecular functions induced by Emdogain are mediated via TGF-βR1, in particular ALK-5 signaling.

However, there are still many open questions. Is there one or more molecules in Emdogain that work via TGF-βRI kinase? Is it possible that the activation of TGF-βRI is caused by cross activation and the initial response comes from another receptor such as epidermal growth factor receptor [53], insulin-like growth factor receptor [54], BMP signaling [55], Wnt signaling [56], or integrins [57]? To what extent do other cell types exert their response to Emdogain via TGF-βRI kinase? Does Emdogain behave similarly in vivo, considering the implication of protease and cofactors? Important also is the question of the clinical relevance of the present finding.

The clinical relevance of the present observations can be based on the assumption that Emdogain causes cellular responses similar to pleiotropic TGF-β. Besides what is known from genetic models [58], [59], the local application of recombinant TGF-β causes the formation of a collagen-rich tissue, for example in the skeletal muscle [60]. These findings are consistent with the role of Emdogain in supporting the formation of a collagen matrix in porcine skin wound healing models [3]. It is thus reasonable to assume that Emdogain and recombinant TGF-β are similar in their ability to stimulate the formation of a collagen-rich extracellular matrix. Additional work is required to determine to which extent, and if at all, the in vivo response to Emdogain is mediated by ligands stimulating TGF-β receptor signaling in the respective target cell.

In summary, our findings suggest that the in vitro cellular response of palatal fibroblasts to Emdogain exclusively depends on TGF-βRI kinase signaling. It will now be important to determine why the genetic response of >100 genes in fibroblasts to the complex composition of the xenogeneic preparation of enamel matrix derivative exclusively depends on the bottleneck of TGF-β receptor signaling. The complexity of the composition of the porcine enamel matrix derivative is highlighted by the chromatography [1] and proteomics [61]. Our unexpected observation that Emdogain-regulated gene expression in palatal fibroblasts strictly requires TGF-βRI kinase signaling opens the door for research on the respective ligands and other potential target cells.
